# Supplementary figures and images for: Loss of Tsc1 in cerebellar Purkinje cells induces transcriptional and translation changes in FMRP target transcripts
Source: eLife. 2021 Jul 14;10:e67399. doi: 10.7554/eLife.67399 (PMC8279760; doi:10.7554/eLife.67399)

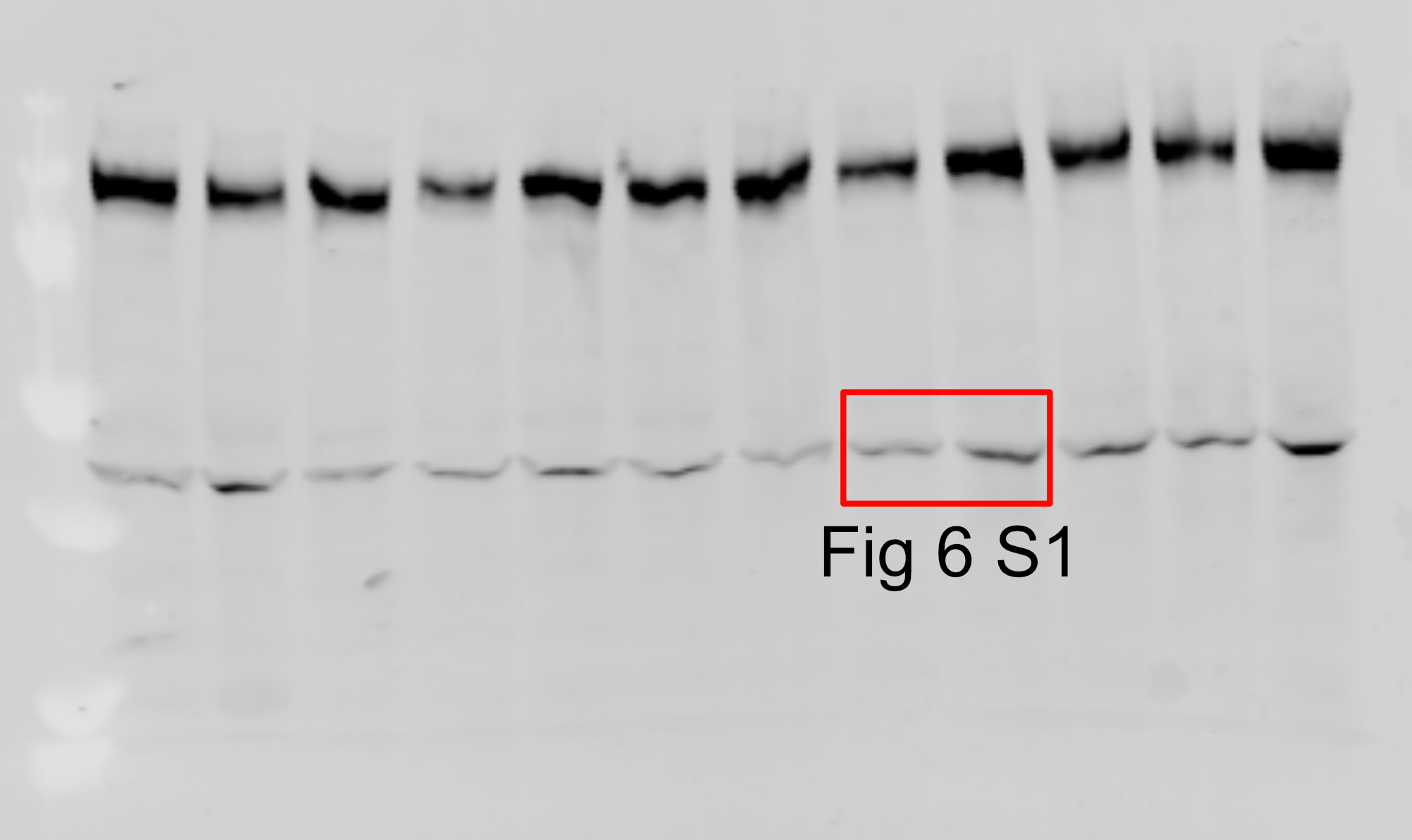

Supplement: Source data 1. [file elife-67399-data1.zip › bactin_1_labeled.tiff]

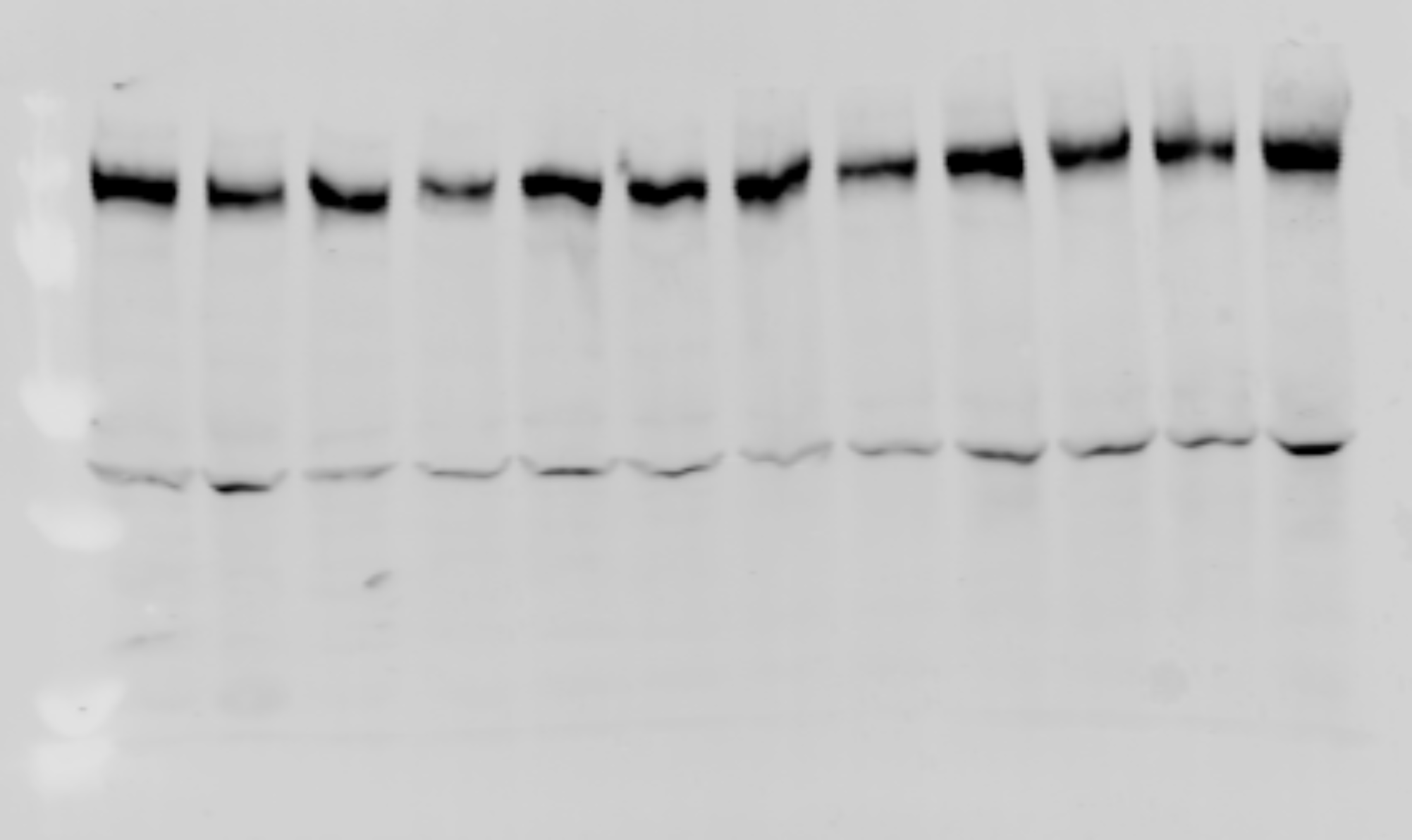

Supplement: Source data 1. [file elife-67399-data1.zip › bactin_1.tif]

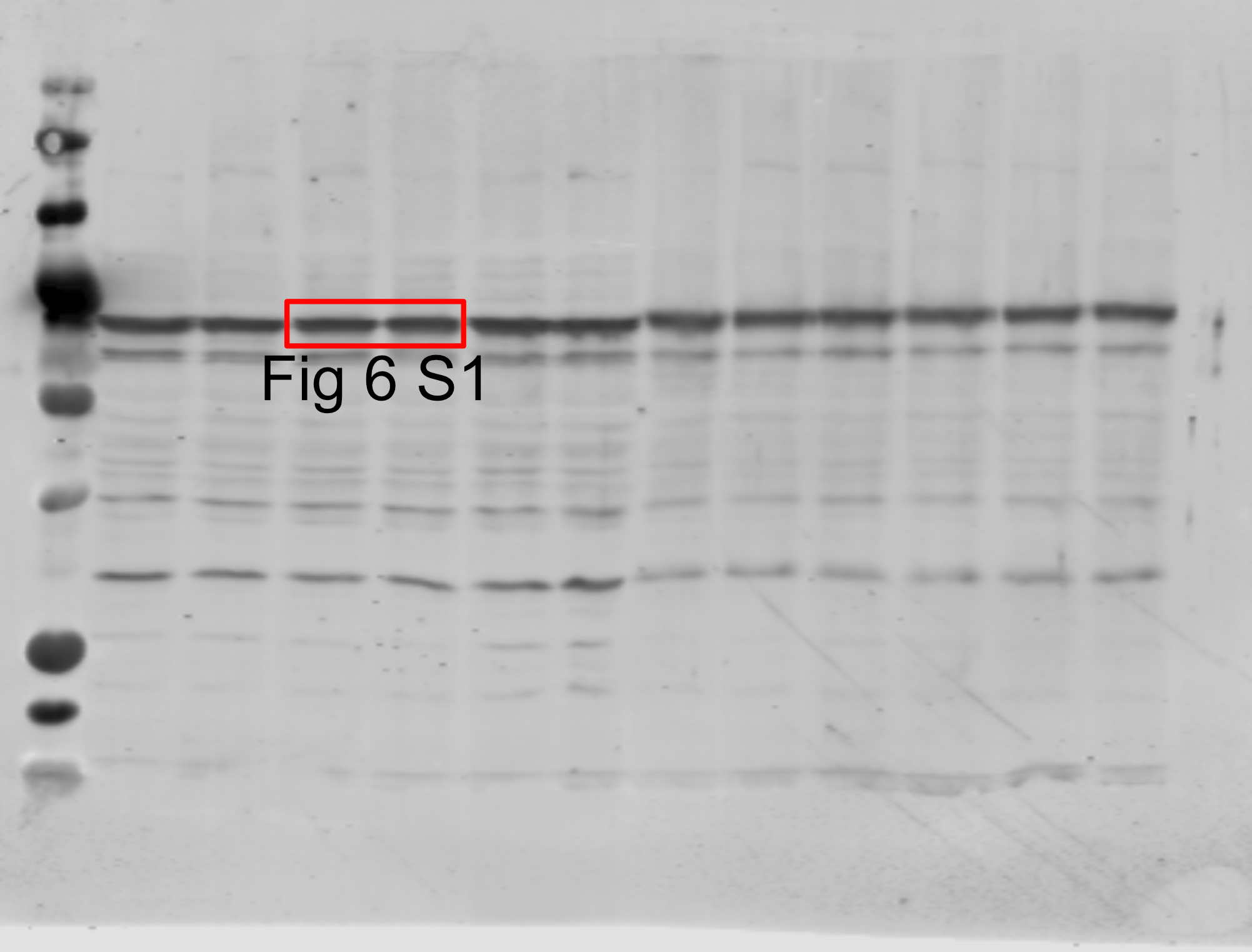

Supplement: Source data 1. [file elife-67399-data1.zip › Cdk16_2_labeled.tiff]

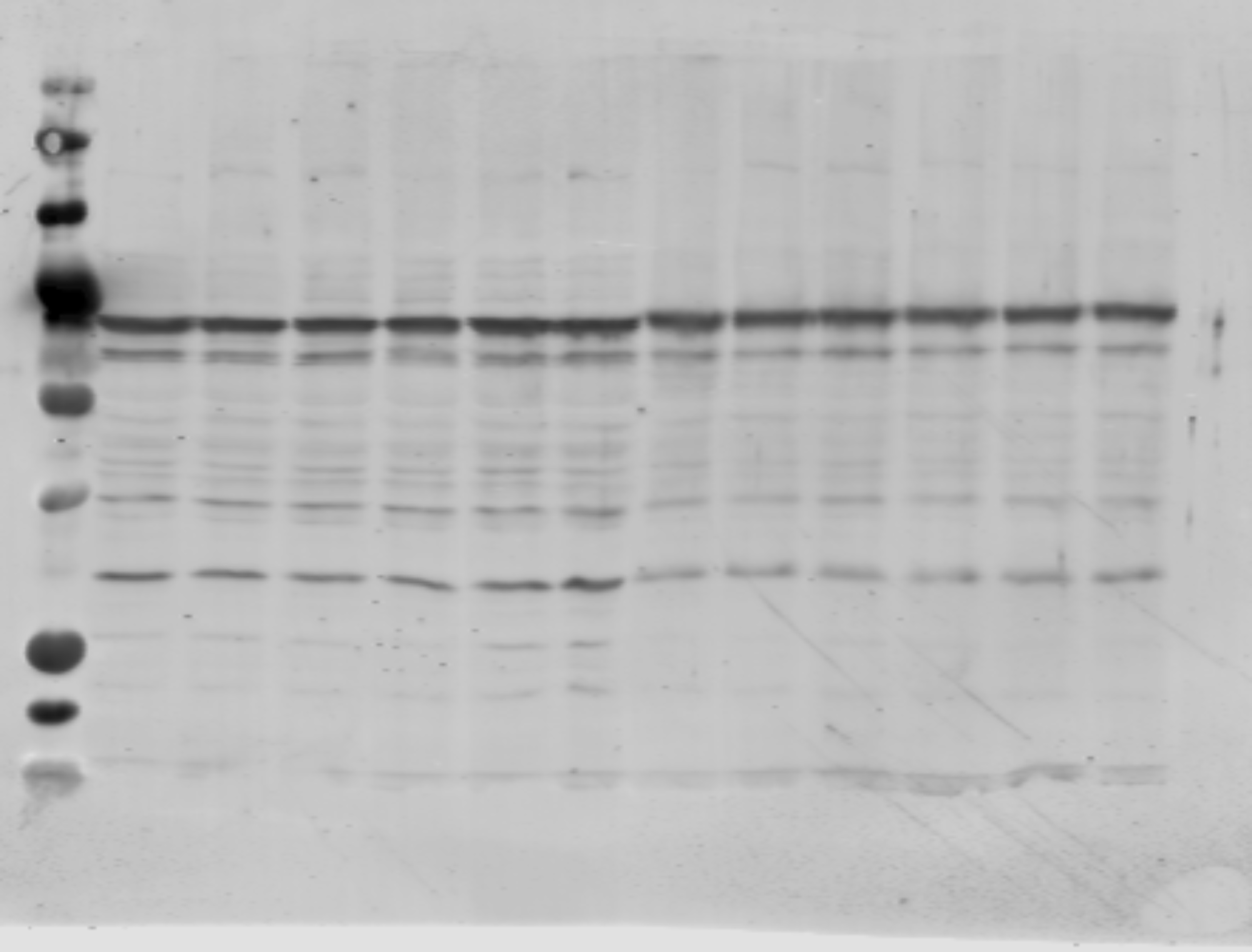

Supplement: Source data 1. [file elife-67399-data1.zip › Cdk16_2.tif]

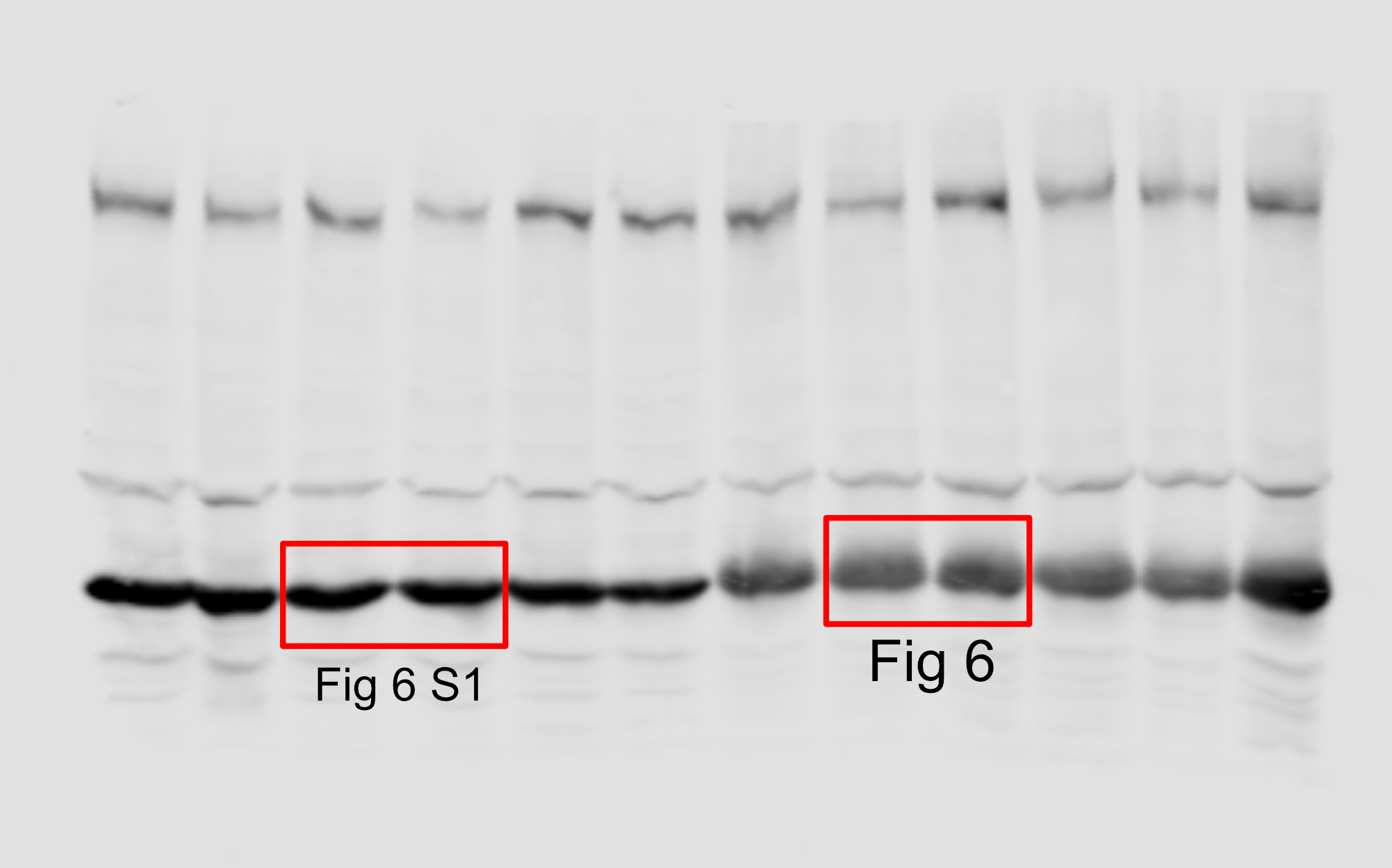

Supplement: Source data 1. [file elife-67399-data1.zip › gapdh_1_labeled.tiff]

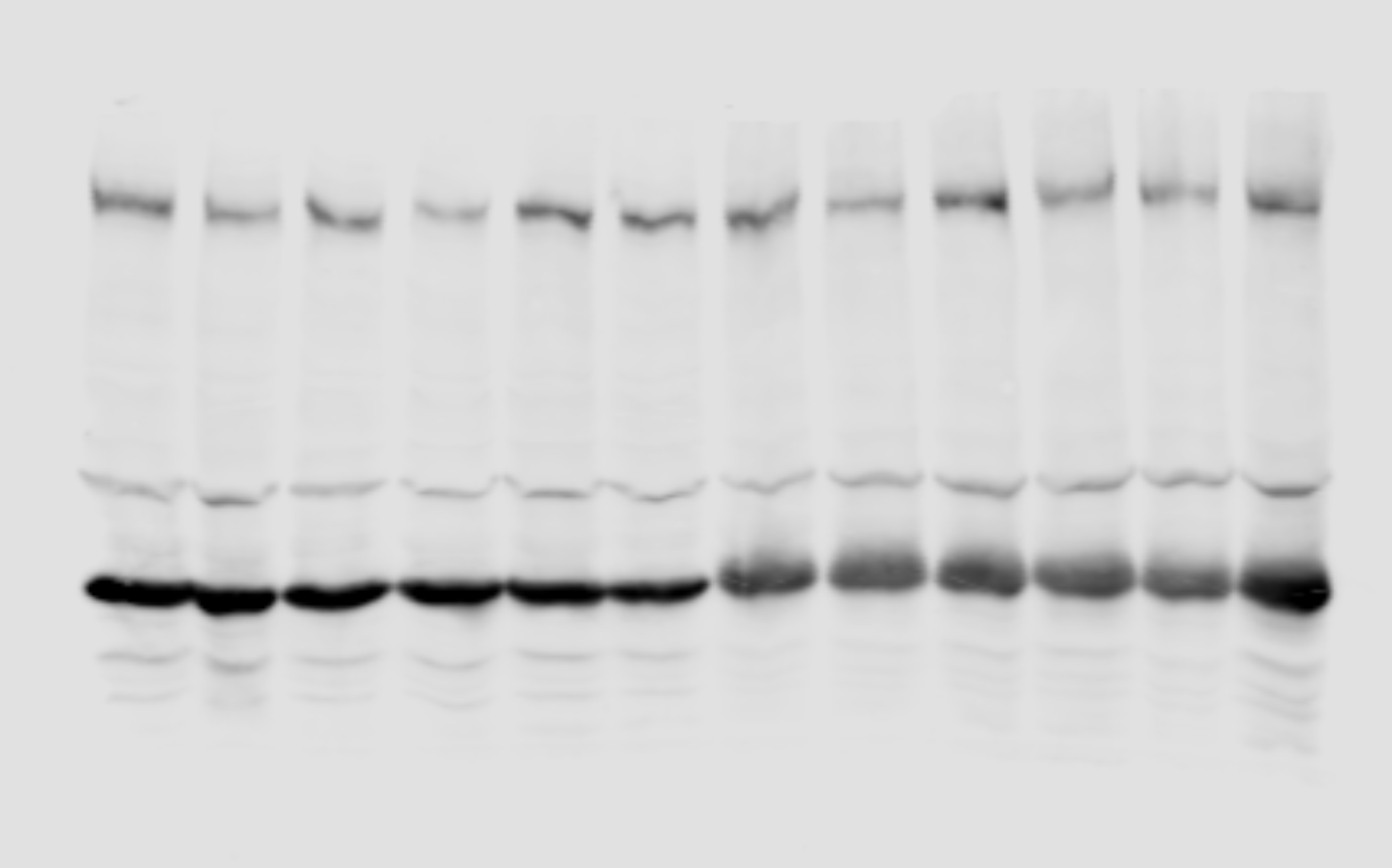

Supplement: Source data 1. [file elife-67399-data1.zip › gapdh_1.tif]

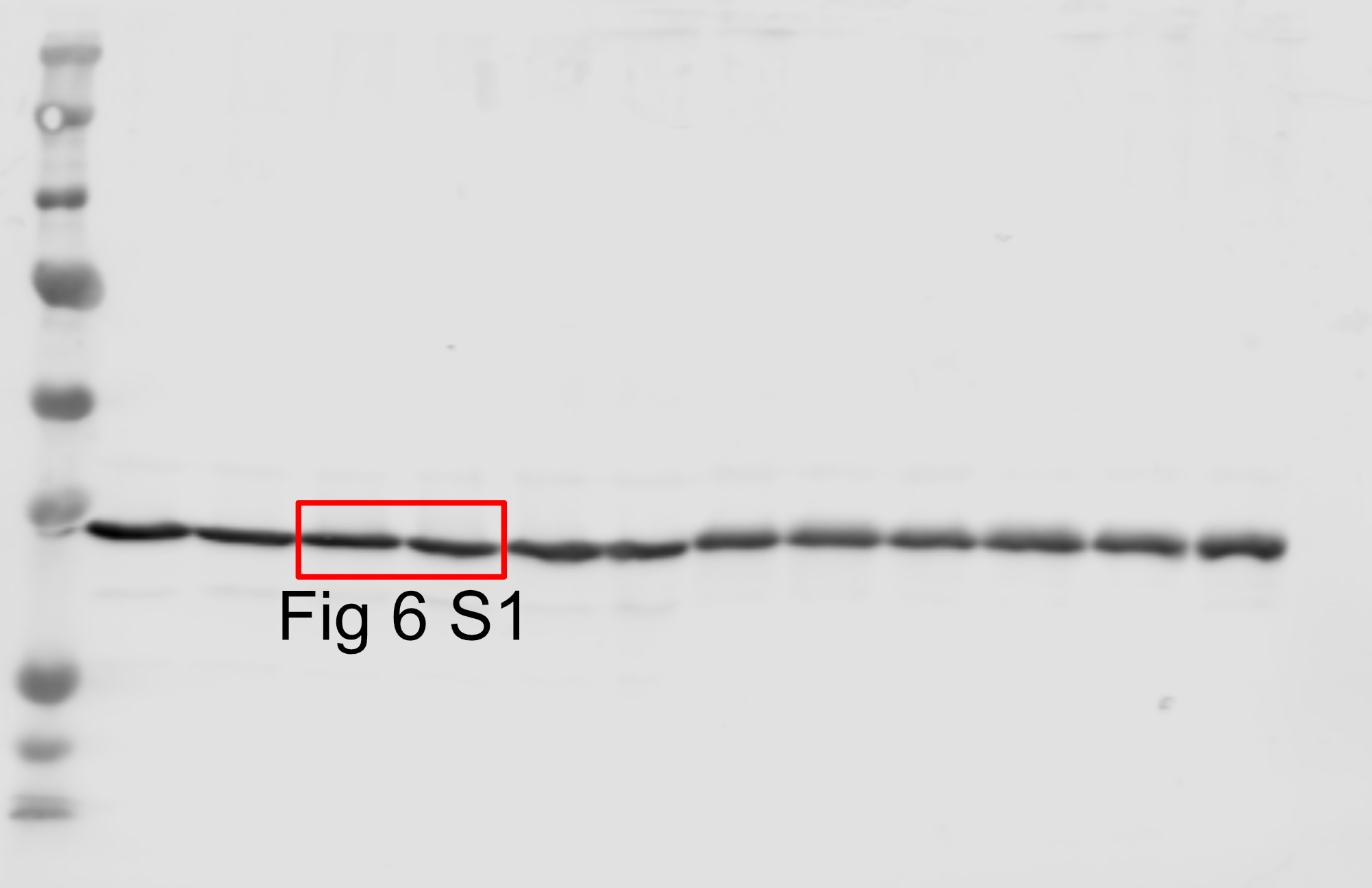

Supplement: Source data 1. [file elife-67399-data1.zip › Gapdh_2_labeled.tiff]

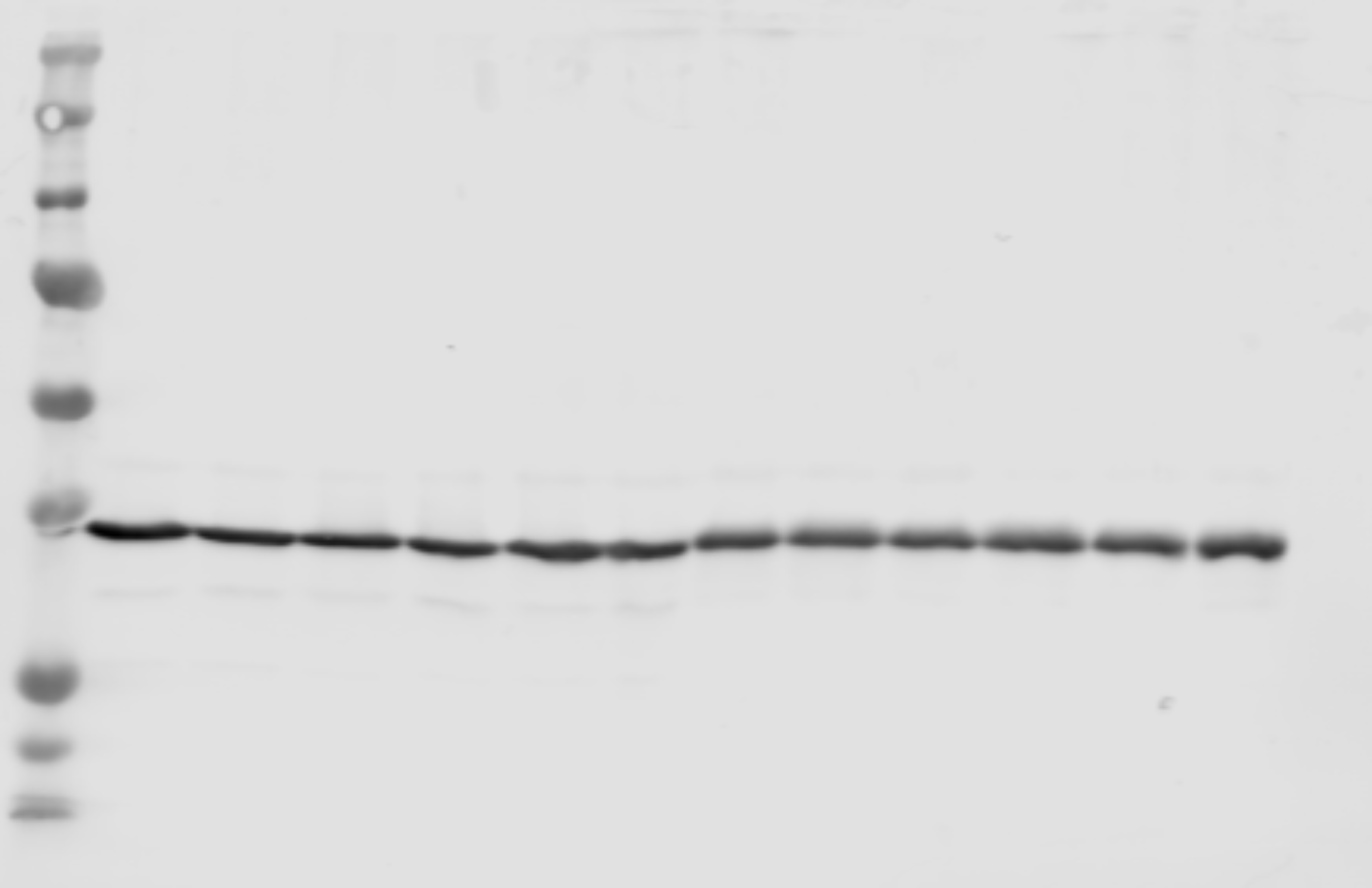

Supplement: Source data 1. [file elife-67399-data1.zip › Gapdh_2.tif]

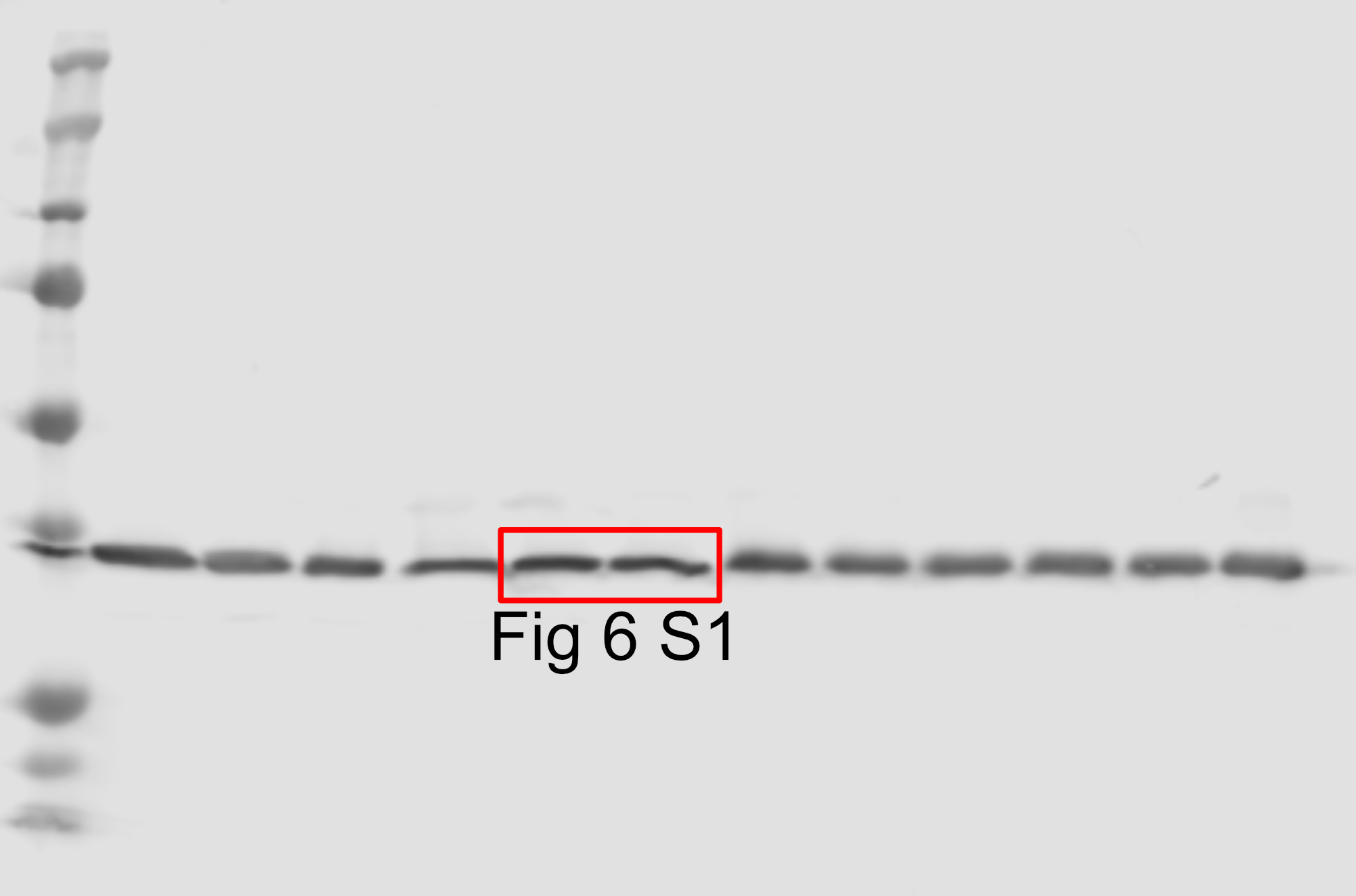

Supplement: Source data 1. [file elife-67399-data1.zip › Gapdh_3_labeled.tiff]

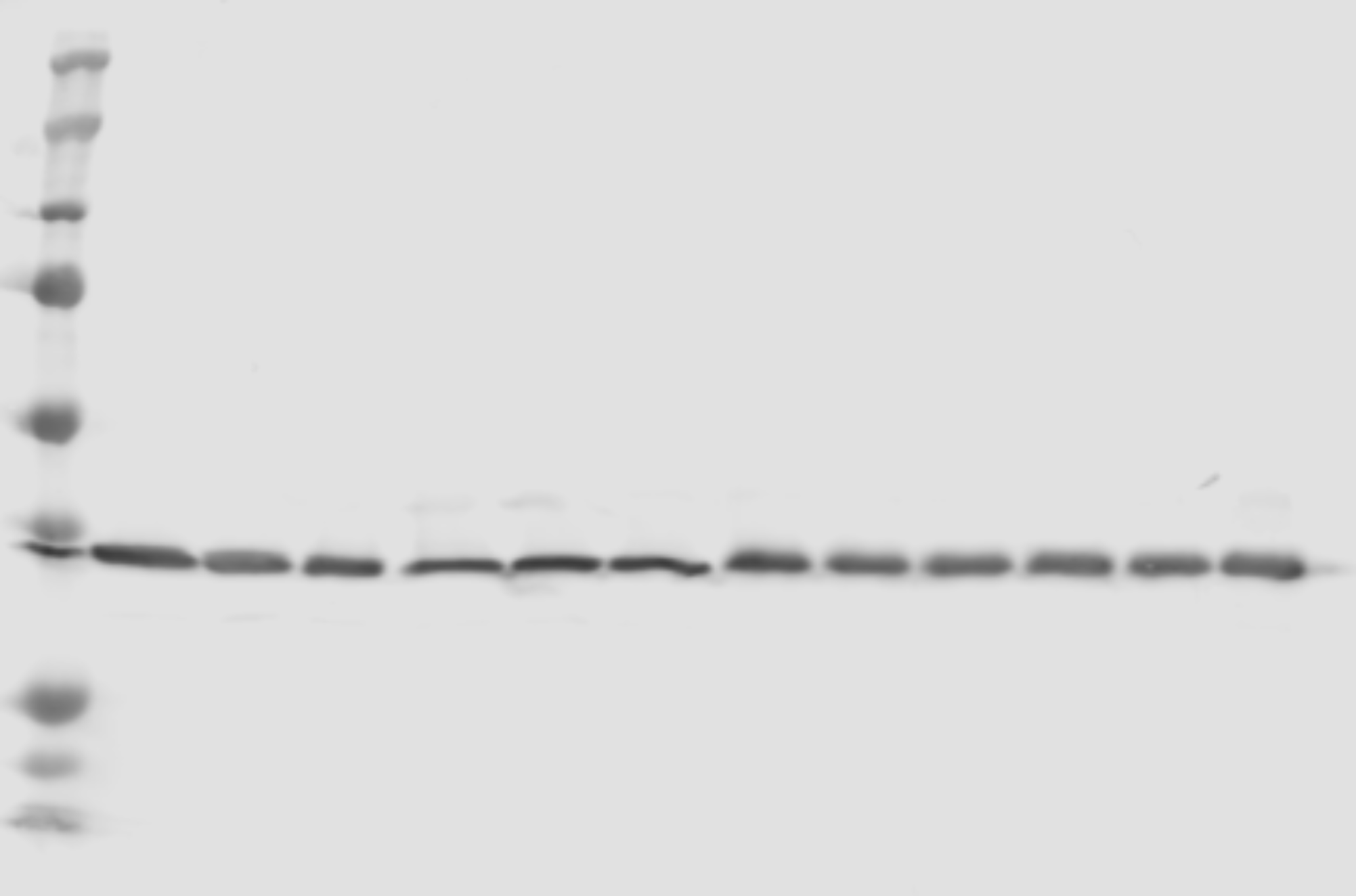

Supplement: Source data 1. [file elife-67399-data1.zip › Gapdh_3.tif]

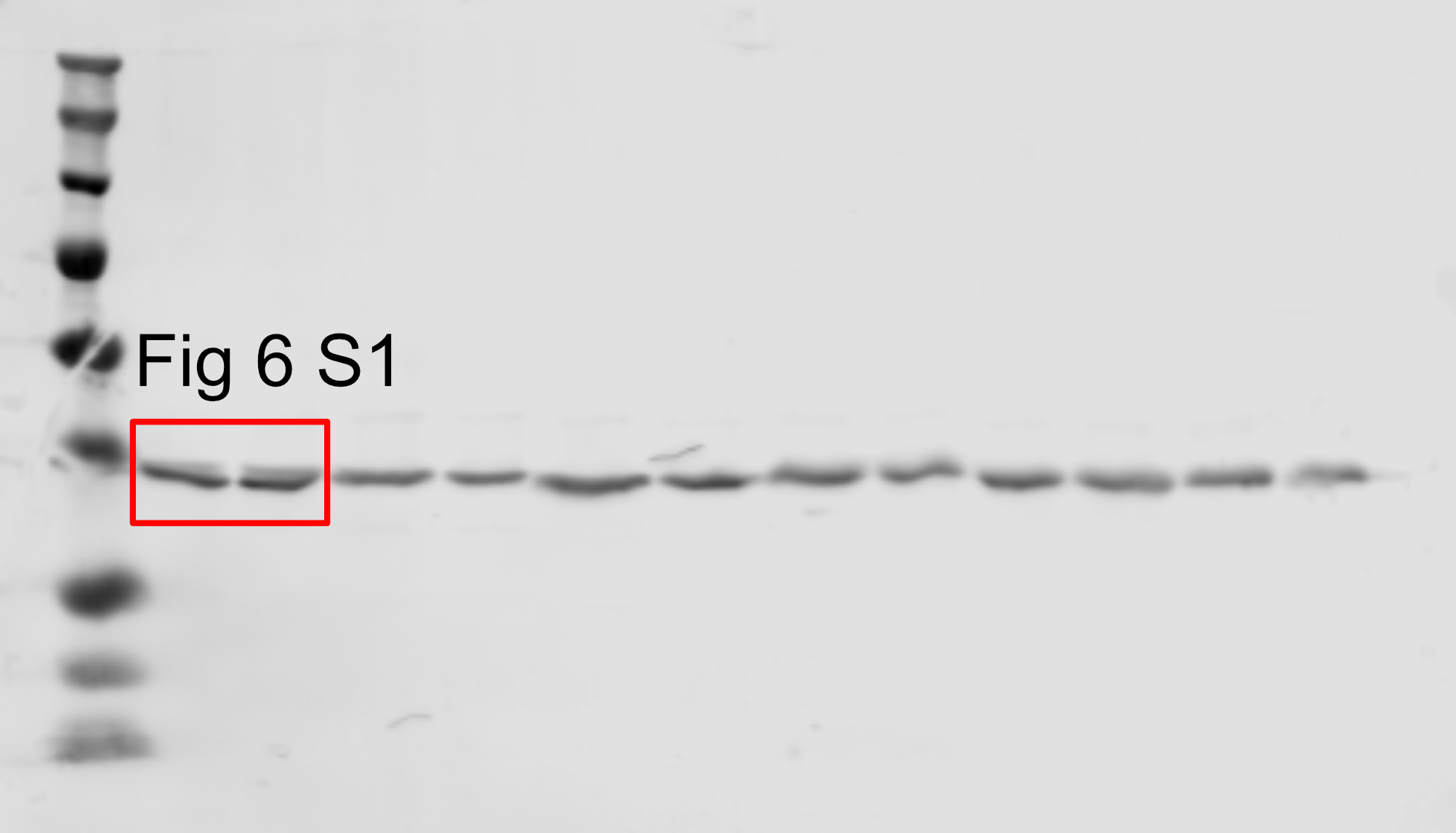

Supplement: Source data 1. [file elife-67399-data1.zip › Gapdh_4_labeled.tiff]

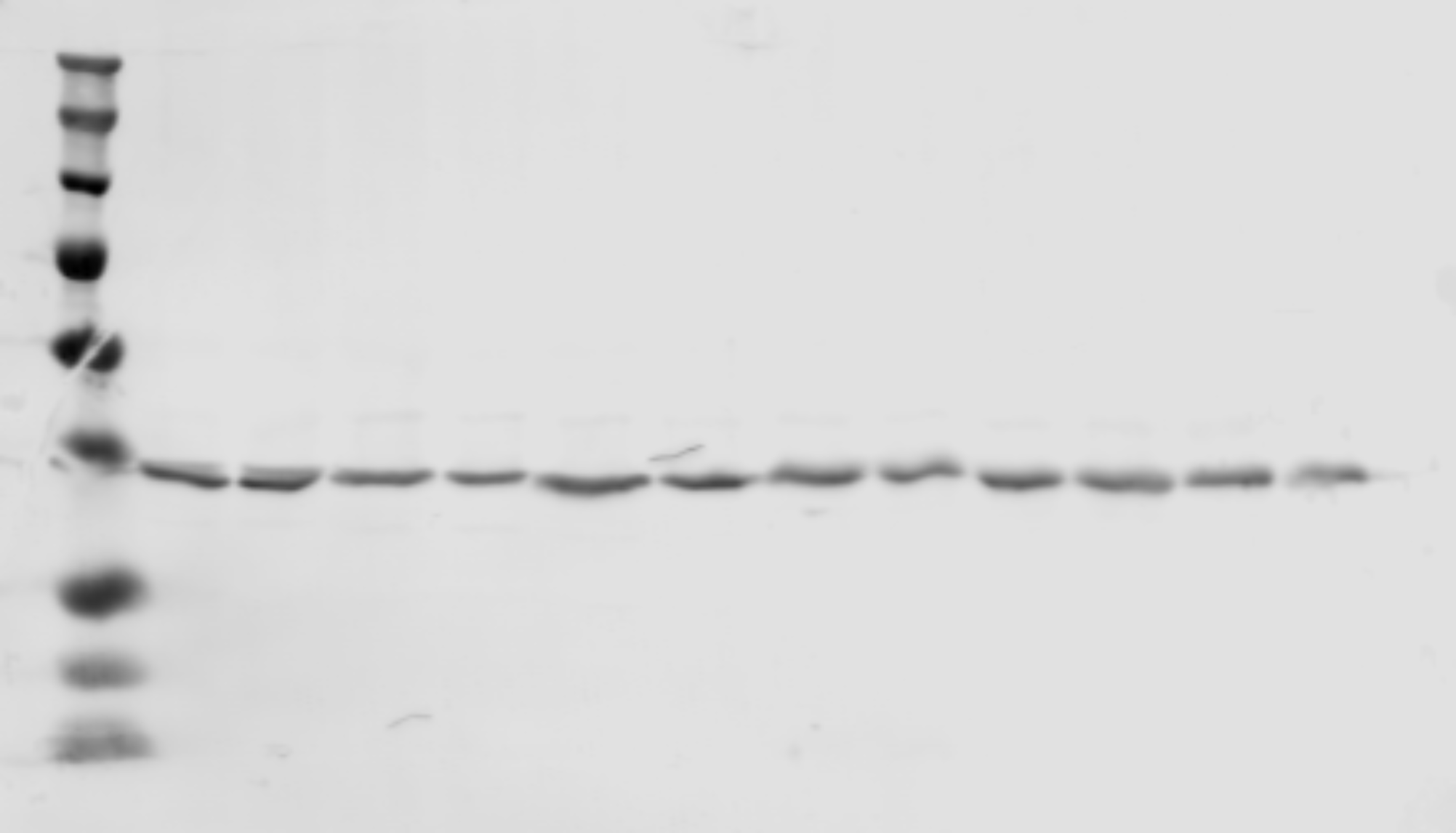

Supplement: Source data 1. [file elife-67399-data1.zip › Gapdh_4.tif]

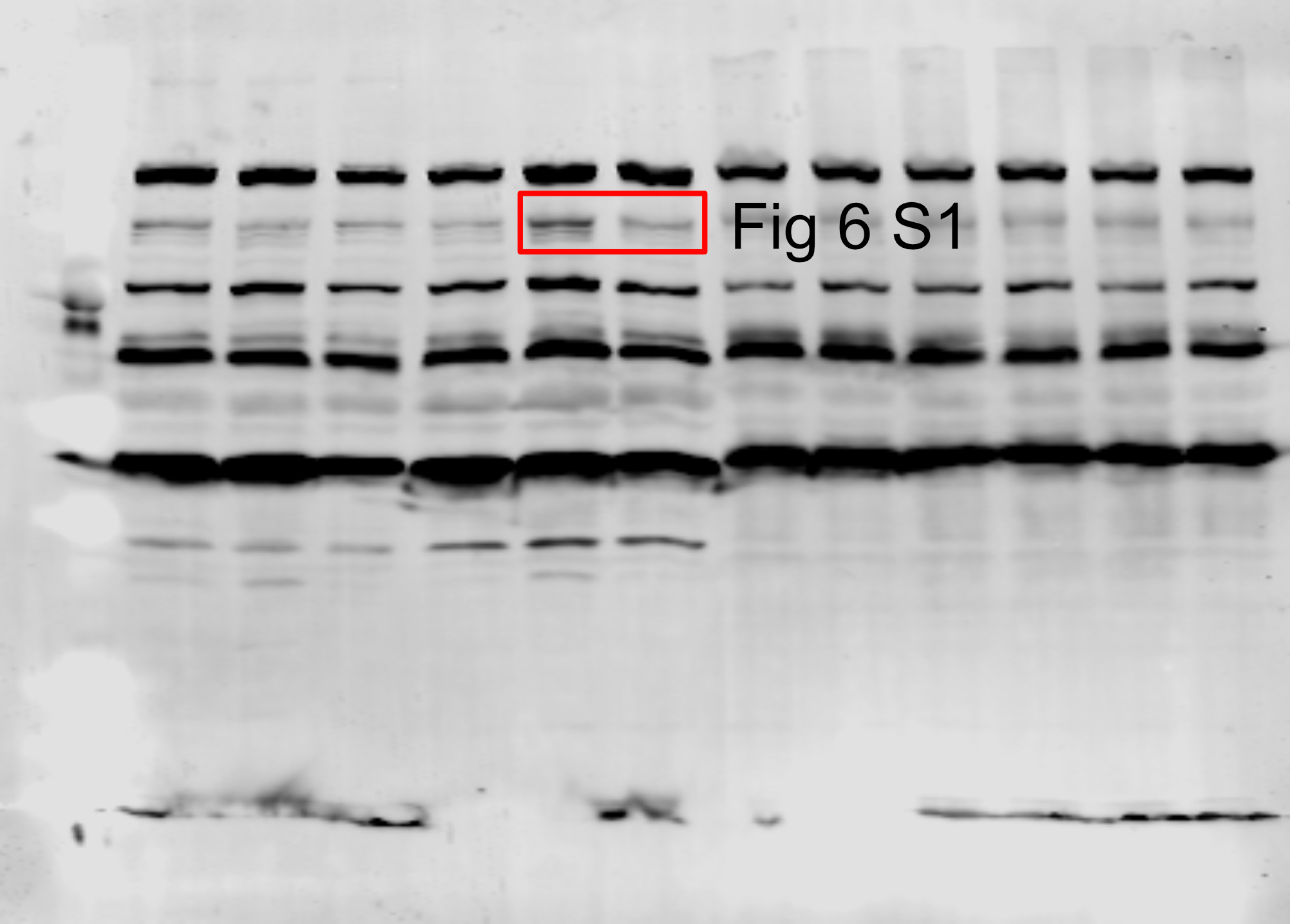

Supplement: Source data 1. [file elife-67399-data1.zip › Kif3c_3_labeled.tiff]

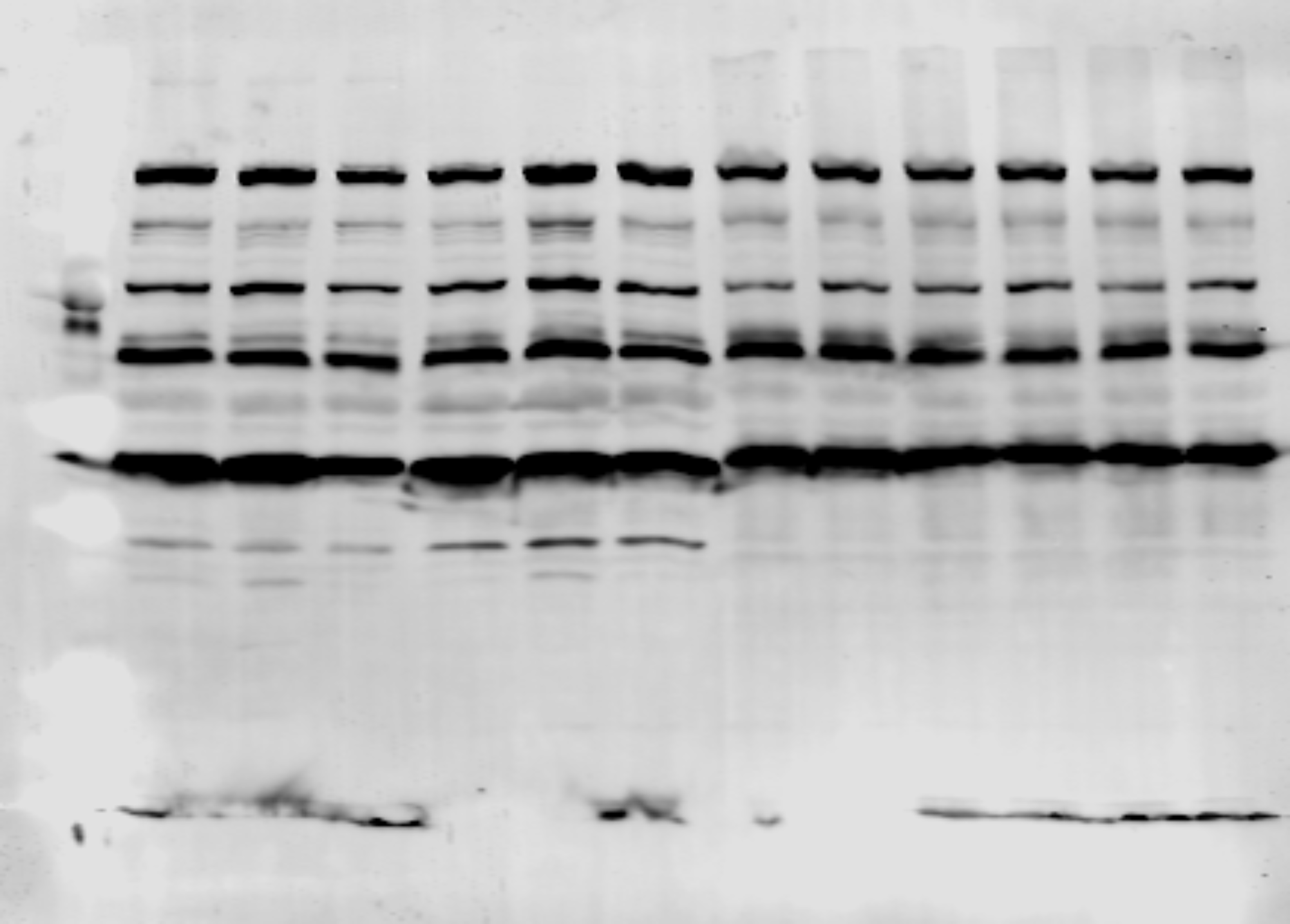

Supplement: Source data 1. [file elife-67399-data1.zip › Kif3c_3.tif]

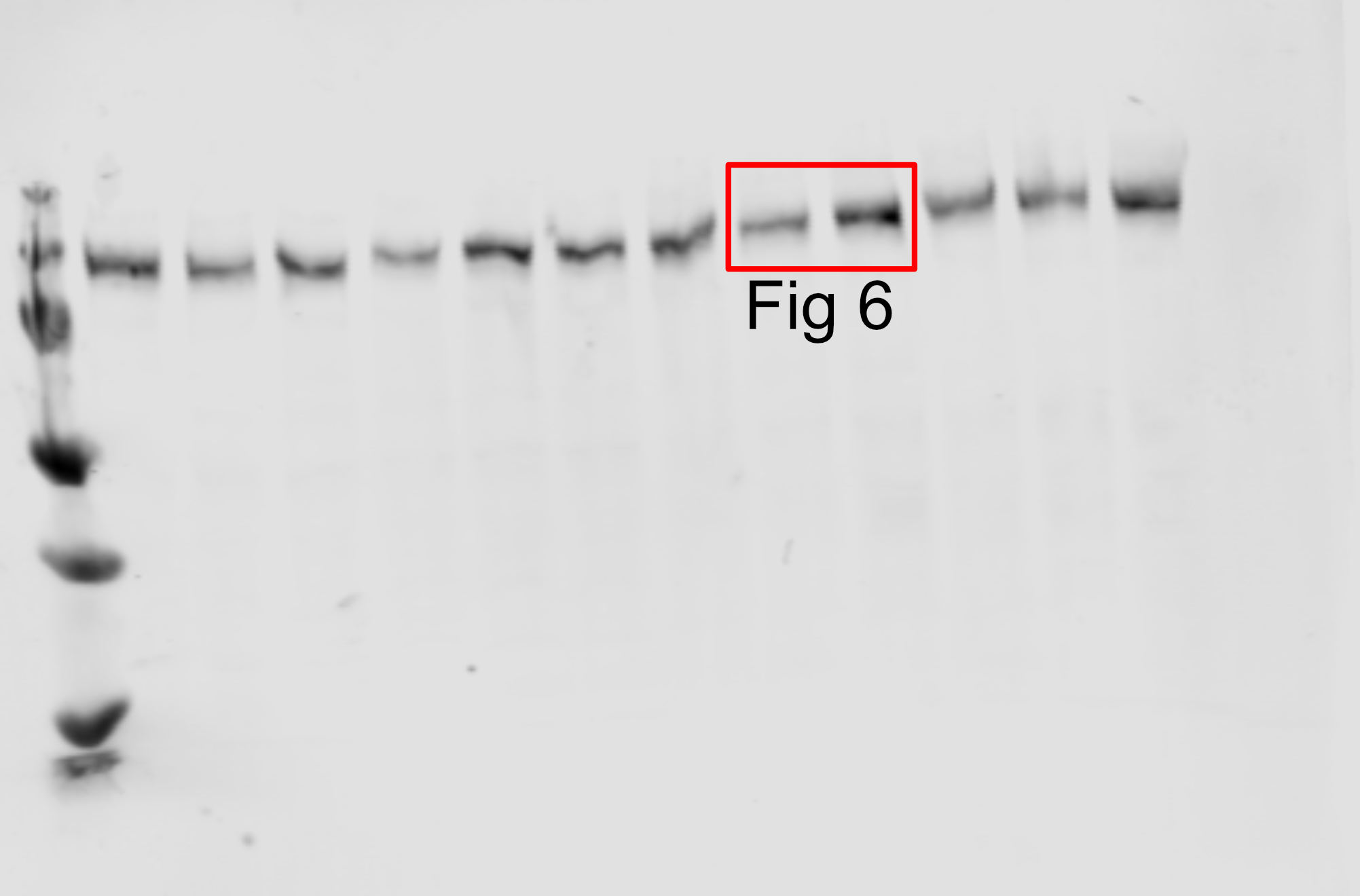

Supplement: Source data 1. [file elife-67399-data1.zip › psd93_labeled.tiff]

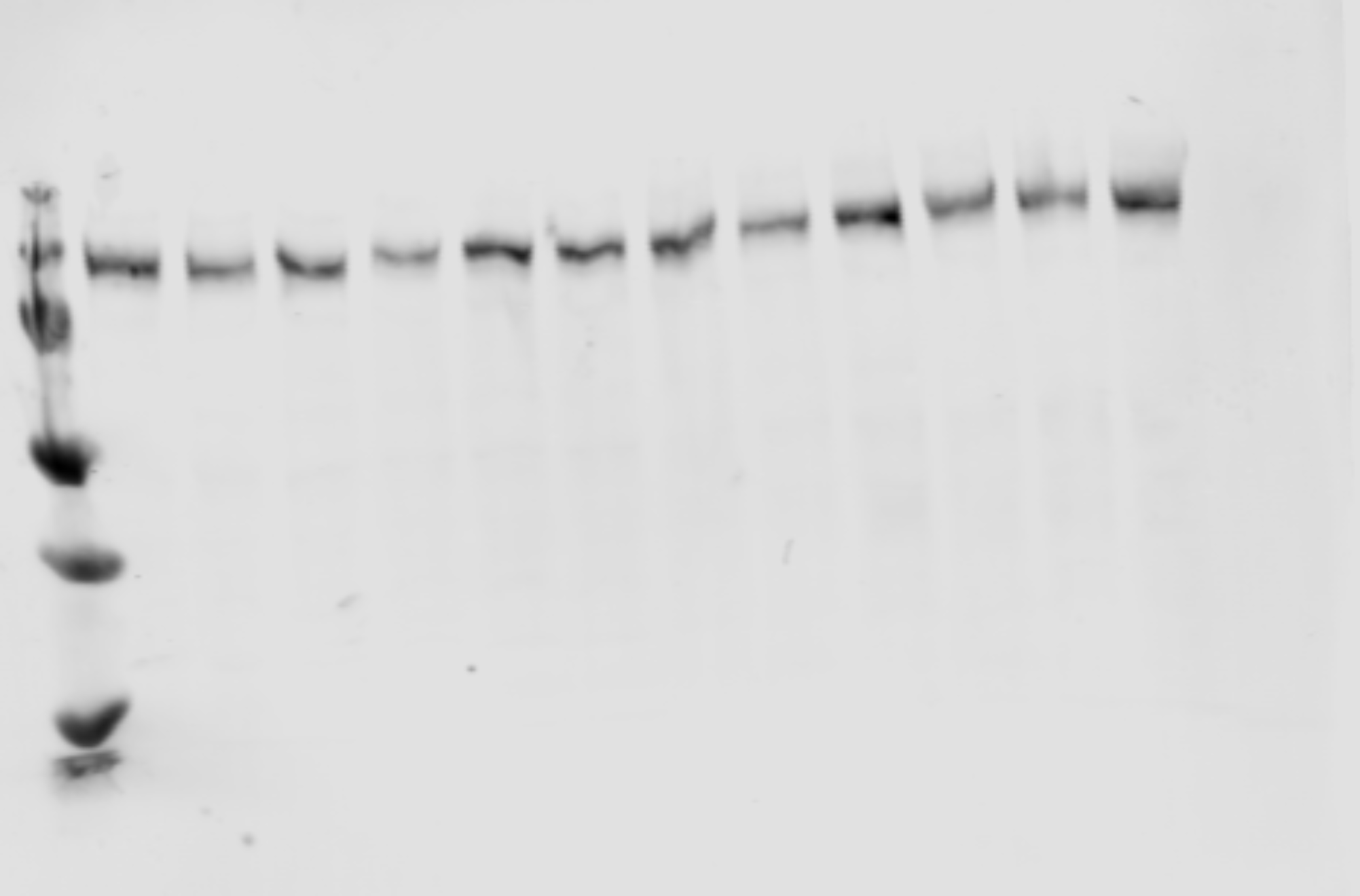

Supplement: Source data 1. [file elife-67399-data1.zip › psd93.tif]

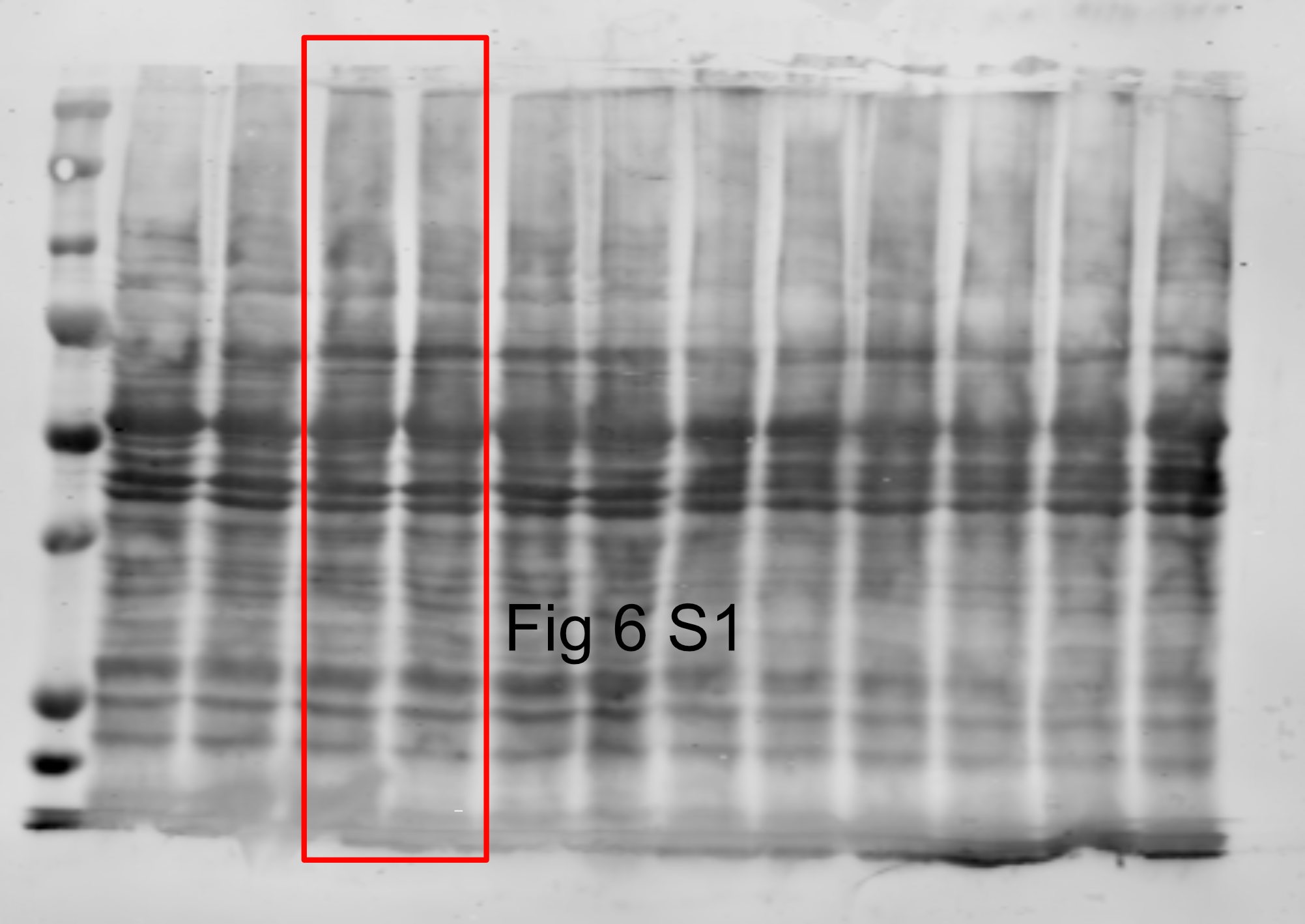

Supplement: Source data 1. [file elife-67399-data1.zip › total_protein_2_labeled.tiff]

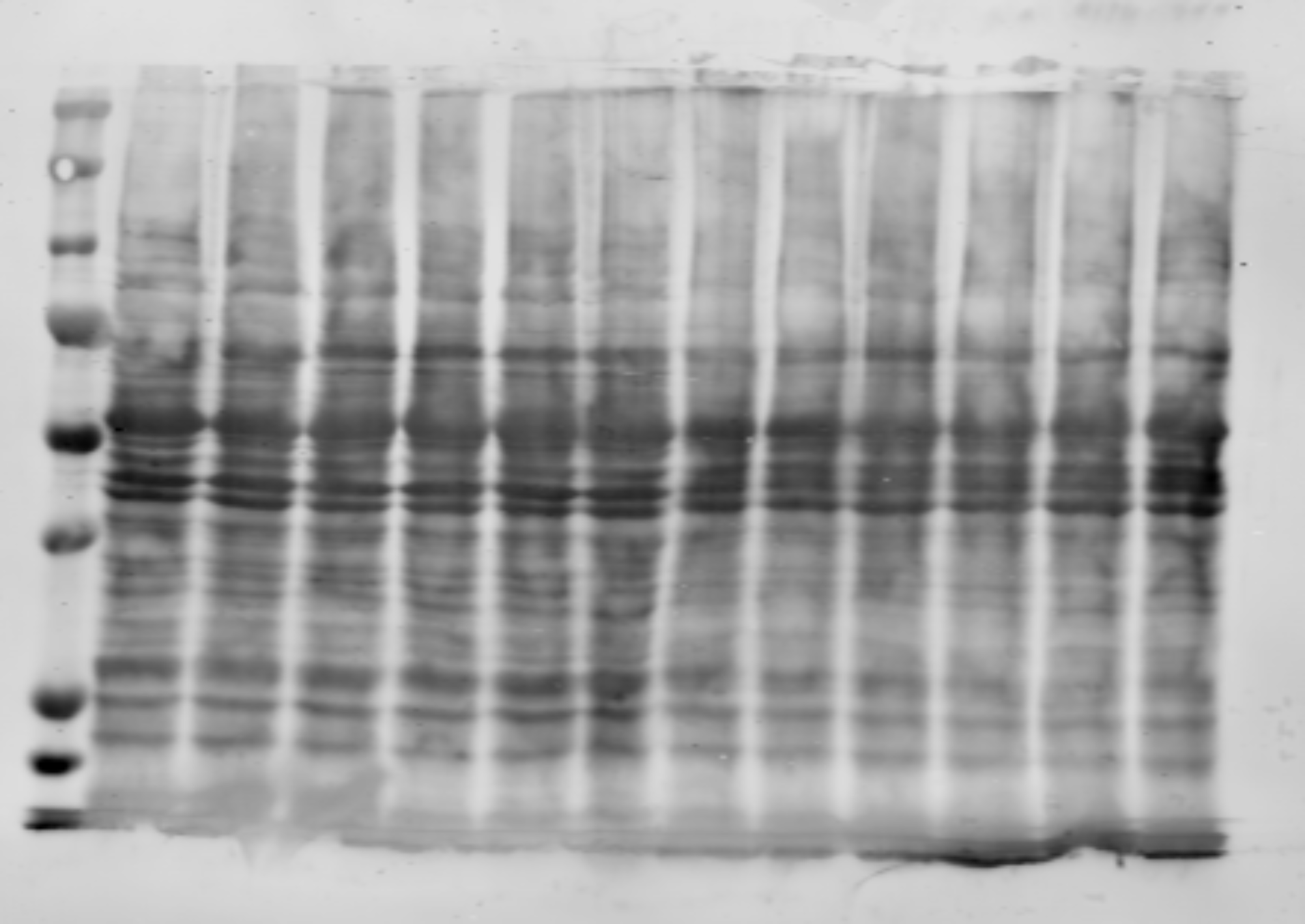

Supplement: Source data 1. [file elife-67399-data1.zip › total_protein_2.tif]
